# Case of Apoplexia Hydrocephalica

**Published:** 1802-07

**Authors:** 

**Affiliations:** Ansford, near Castle Cary


### *Case of Apoplexia Hydrocephalica. Communicated by* Dr. WOODFORDE, *of Ansford, near Castle Cary.*

HARRIET WOODFORDE, ætat. 10. Dec. 23, 1801, complains of conſtant heavineſs and pain in the anterior part and left ſide of the head, much increaſed on ſtooping. The veſſels of the tunica adnata in both eyes are very red and turgid; pulſe 120, and ſomewhat hard; ſkin hot; tongue almoſt natural; little thirſt; one ſtool yeſterday. Laſt night ſhe awoke ſuddenly, with much agitation and great complaint of pain in her head, and ſaid, that near objects appeared diſtant. The pain has been preſent three days, and on its acceſſion was more general. It ſupervened to acute pain and ſtiffneſs in the poſterior part of the neck, which came on ſuddenly, and continued, with ſlight remiſſions, a fortnight. Applicentur temporibus hirudines quatuor. & fiat v. s. ad ℥vj. ℞. Pulv. rhei. gr. vj. Calomel. gr. iij. f. p. s. s. In the evening, I found the pain was diminiſhed, and that the leeching and bleeding had ſucceeded well. Blood very florid, and with a ſmall proportion of ſerum. No buffy coat; but it muſt be remarked, that the blood ran down the arm, and that ſhe was fearful of the operation. Three or four copious ſtools from the phyſic.

℞. Miſtur. ſalin. ℥j. ſal. nitri. gr. vj. Mucil. g. Arab. ſyr. balſ. aa. ʒj. tinct. digital. gt. iv. M. ut fiat hauſtus 4tis horis ſumendus. Pulſe, whilſt aſleep, 100, and rather full.

24th, 10 A. M. Has paſſed a good night, and is free from pain, except on ſtooping. Eyes ſtill inflamed. P. 100. No nauſea. Repetatur hauſtus c. gt. v. tinct. digital.

Hora

Hora 8va. P. M. Continues free from pain; eyes lefs inflamed, and can bear ftooping much better. One ftool to-day. Repetatur hauftus c. gt. vj. 8vis horis. P. only 73, fk. cool, app. good.

25th, 10 A. M. Has had a good night, and is without any pain. Eyes rather more inflamed, but feels no inconvenience from ftooping. P. 100, t. fk. and b. natural. Continuantur M. ut heri.

27th, 10 A. M. Pulfe 88, and fenfibly a little intermittent. Slight and tranfient return of pain in the forehead laft night; none fince. Eyes ftill inflamed. Three ftools yefterday. Fell afleep laft evening, and upon awaking complained again that near objects appeared at fome diftance; pupils not dilated; vifion good fince. Repetatur hauft. bis tantum in die c. gt. iv.

31ft, No return of pain of head nor diftant vifion; yefterday complained of violent aching pain in the left leg, which lafted for feveral hours. Eyes ftill red and turgid; fk. t. and b. natural; p. 100. Has taken the tinct. digital. only twice a day; and this morning, by miftake, only four drops. Capiet T. digital. gtt. vj. 4ter die et etiam pulv. cinchon. gr. x. bis die.

Jan. 2. Pulfe 80, and intermittent; eyes lefs red; no return of pain of head; fome pain yefterday in the knees; app. good; bowels regular. Contin.

I was immediately and forcibly ftruck with the violence and fudden acceffion of the above fymptoms, and was led to conclude them to be the incipient ones of that dreadful and generally fatal difeafe denominated by Doctor Cullen the Apoplexia Hydrocephalica. I had alfo a more accurate and ready apprehenfion of the difeafe, from the indelible impreffion made on my mind by the death of her fifter, three years fince, at the age of eighteen, after labouring three weeks under every characteriftic fymptom of the fame. In this cafe, early and proper treatment was unfortunately omitted; I faw her on the fourteenth day, a period too late for fuccefs. In the prefent, a fpeedy and decifive practice flattered my expectation; and I need not mention the heart-felt pleafure experienced on the happy event.

Many cafes may be adduced from others, in teftimony of the fuccefs attending in this difeafe the early ufe of bleeding, both general and topical, with cathartics, digitalis, and the rigid obfervance of every part of the antiphlogiftic regimen. Our knowledge of the juvantia throws much light on the nature and caufe of the difeafe. It appears to me, that either active inflammation, or local plenitude, producing what may be called chronic inflammation, is in almoft every inftance the proximate caufe; and that the effufion into the ventricles is only an effect of of previous turgefcence, or increafed action in the vafcular fyftem; and that the only means of faving the patient is by removing the firft ftate, and thus obviating or preventing the fecond. To anfwer thefe intentions, in conjunction with bleeding, cathartics, and a proper regimen, I anticipate the great and falutary powers that will henceforth be derived from that potent auxiliary digitalis. It is in this and fimilar difeafes, depending on or connected with increafed action in the fanguiferous fyftem, that both reafon and experience teach us the efficacy of this remedy. In fome other difeafes, and efpecially in phthifis pulmonalis, a fanguine temper, inexperience, and enthufiafm, have led many of its advocates greatly to over-rate its virtues; and I think, from the difappointment thus occafioned, real injury has been done to the reputation of the medicine. Notwithftanding all that has been faid and done in the cure of phthifis, who can yet ftep forward and affert, from long and unequivocal experience, that digitalis operates in it fpecifically, or fimilar to the Peruvian bark in the cure of intermittents?

*February* 1, 1802.

